# Idiopathic Hypoparathyroidism in an Osteoporosis Patient Receiving Denosumab Therapy

**DOI:** 10.7759/cureus.102570

**Published:** 2026-01-29

**Authors:** Louie S Yang, Darcy S Tokunaga, Sian Yik Lim

**Affiliations:** 1 Family Medicine, Straub Benioff Mililani Clinic, Mililani, USA; 2 Medicine, University of Hawaii John A. Burns School of Medicine, Honolulu, USA; 3 Rheumatology, Pali Momi Medical Center, Aiea, USA

**Keywords:** case report, denosumab, hypoparathyroid, idiopathic hypoparathyroidism, osteoporosis, pth abnormalities, s: hypocalcemia

## Abstract

Idiopathic hypoparathyroidism is a rare endocrine disorder characterized by impaired parathyroid hormone (PTH) secretion leading to hypocalcemia and hyperphosphatemia. We report the case of a 71-year-old female patient receiving denosumab for osteoporosis who was found to have asymptomatic hypocalcemia prior to her fifth denosumab injection. Lab results revealed a low ionized calcium level of 4.45 mg/dL, a low normal total calcium level of 8.2 mg/dL, and an inappropriately low PTH level of 17 pg/mL, with normal magnesium and vitamin D levels. Calcium levels were normal during the previous four doses. With no history of neck surgery, renal disease, autoimmune disease, or malabsorption, a diagnosis of idiopathic hypoparathyroidism was supported, likely unmasked by denosumab-associated suppression of bone resorption. Denosumab treatment was withheld, and calcium carbonate and calcitriol supplementation led to stabilization of serum calcium in the low-normal range, but PTH levels remained suppressed. Our case highlights the importance of ensuring normal calcium levels before denosumab administration and emphasizes the challenges of osteoporosis management in patients with hypoparathyroidism. In this area of treatment, evidence-based data remains limited.

## Introduction

In the United States, hypoparathyroidism has an estimated prevalence of 37 cases per 100,000 person-years [1]. Of those impacted by hypoparathyroidism, approximately 75% of cases are postsurgical, with the remainder due to genetic, autoimmune, or idiopathic causes [2]. Idiopathic hypoparathyroidism refers to hypoparathyroidism without an identifiable cause, occurring when the parathyroid glands fail to produce adequate parathyroid hormone (PTH) in the absence of known etiologies such as surgical removal, autoimmune destruction, or genetic disorders [1]. PTH plays a central role in maintaining calcium and phosphate balance. Its effects are exerted directly on bone and the kidneys, while its influence on the gastrointestinal tract is indirect, mediated through stimulation of renal production of 1,25-dihydroxyvitamin D (calcitriol) [2]. Calcitriol increases the bodyʻs calcium and phosphate levels by increasing intestinal absorption, promoting bone mineralization, and reducing kidney excretion, while exerting negative feedback on PTH.

Hypoparathyroidism is characterized by low or inappropriately normal PTH levels in the serum, hypocalcemia, and hyperphosphatemia [3]. Clinical manifestations of these biochemical derangements include neuromuscular hyperexcitability due to hypocalcemia, including circumoral numbness, paresthesias, and muscle cramps, which can progress to laryngospasm, bronchospasm, tetany, and seizures. Other symptoms include fatigue, irritability, and personality changes. Severe hypocalcemia can also lead to prolonged QTc intervals, which can progress to angina and cardiomyopathic damage [4]. Hyperphosphatemia plays a key role in promoting abnormal mineral deposition in soft tissues, including the vasculature, brain, kidneys, and other organs [2].

Denosumab is a monoclonal antibody used to treat osteoporosis that binds to the receptor activator of nuclear factor kappa-B ligand (RANKL) and modulates the RANK/RANKL signaling pathway. Under normal physiologic conditions, RANKL binds to RANK receptors on osteoclast precursors and mature osteoclasts to stimulate bone resorption and calcium release from bone. By binding to RANKL, denosumab prevents this interaction, thereby inhibiting osteoclast-mediated bone resorption and turnover and decreasing calcium release from bone [5]. Denosumab can precipitate severe hypocalcemia, especially in patients with risk factors including high baseline levels of bone-specific alkaline phosphatase with >2 areas of bone metastasis, advanced chronic kidney disease (eGFR<30mL/min/1.73m^2), recent thyroid surgery, and concomitant usage of medications that increase calcium loss (e.g., glucocorticoids and some anticonvulsants) [6,7].

It is critical to identify idiopathic hypoparathyroidism in a denosumab-treated osteoporosis patient, as the combination creates a high-risk scenario for severe, prolonged hypocalcemia. Reports of idiopathic hypoparathyroidism associated with denosumab therapy are rare or underdiagnosed, and the optimal strategy for managing osteoporosis in this setting has not been well established. We present a case of a 71-year-old patient in which denosumab osteoporosis treatment unmasked underlying idiopathic hypoparathyroidism, leading to clinically apparent hypocalcemia. We report this case to highlight the need for careful calcium assessment before and after denosumab treatment, due to its possible biochemical implications.

## Case presentation

**Figure 1:**
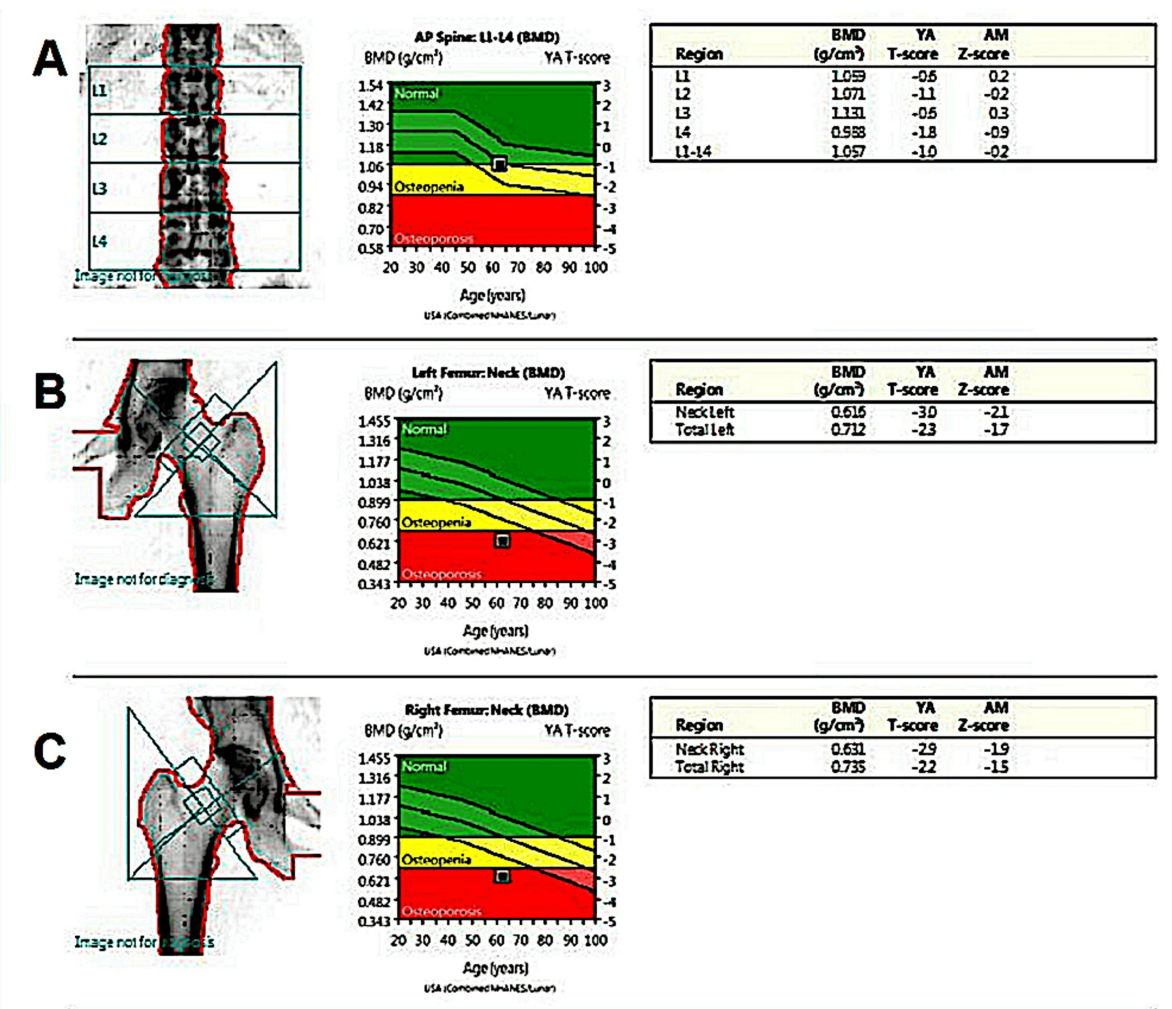
DEXA scan of patient at 63 years old YA: Young adult, AM: Age-matched Lumbar spine T-score of -1.0 (A), left femoral neck of -3.0, left total neck of -2.3 (B), right femoral neck of -2.9, and right total neck of -2.2 (C).

A 71-year-old female patient presents in an outpatient rheumatology clinic for a follow-up of osteoporosis. The patient reported having a total hysterectomy with bilateral salpingo-oophrectomy at age 41 and was started on estradiol supplementation for post-surgical menopause. At age 63, the patient was diagnosed with osteoporosis based on a dual-energy X-ray absorptiometry (DEXA) scan (Figure 1). The DEXA scan showed osteoporotic-range bone mineral density (BMD) at the femoral necks (Figures 1A, 1B) with osteopenia at the lumbar spine and total hips. Baseline laboratory evaluation at the time of osteoporosis diagnosis showed normal serum calcium, phosphate, parathyroid hormone (PTH), magnesium, 25-hydroxyvitamin D, and renal function. Approximately six months after the fourth denosumab dose, routine laboratory testing obtained prior to the scheduled fifth injection revealed hypocalcemia, with a serum calcium level of 6.9 mg/dL (reference range 8.6-10.3 mg/dL).

At age 64, the patient was initially treated with alendronate for osteoporosis; however, the patient reported having stomach discomfort and diarrhea while on this therapy. No formal gastrointestinal workup was pursued, and alternative formulations (effervescent alendronate or intravenous bisphosphonates) were discussed but deferred at that time. Due to the adverse reactions to alendronate, the medication was discontinued, and the patient’s bone density was electively monitored.

**Figure 2:**
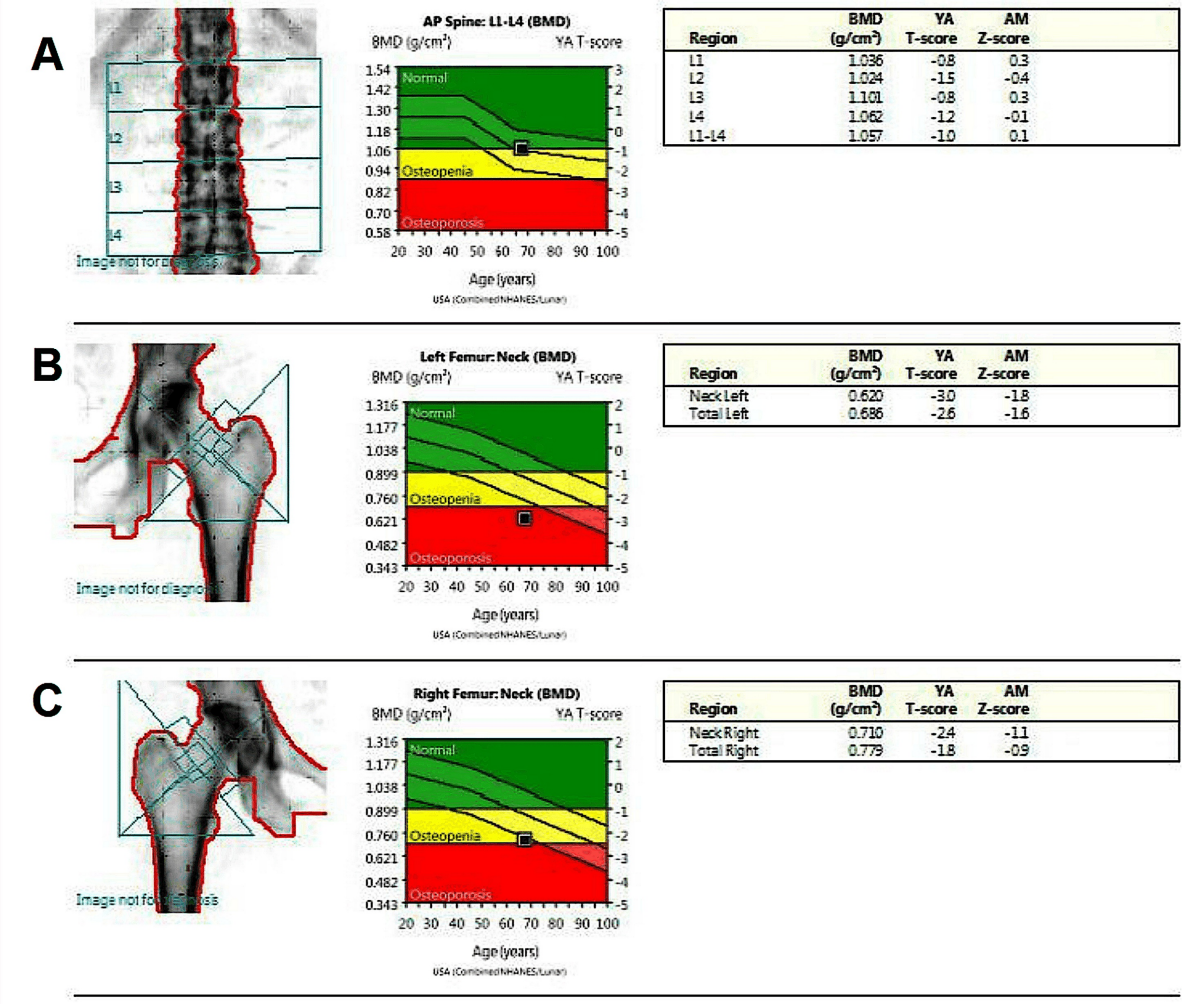
DEXA scan of patient at 67 years old YA: Young adult, AM: Age-matched Lumbar T-score of -1.0 (A), left femoral neck of -3.0, left total neck of -2.6 (B), right femoral neck of -2.4, and right total neck of -1.8 (C)

**Figure 3:**
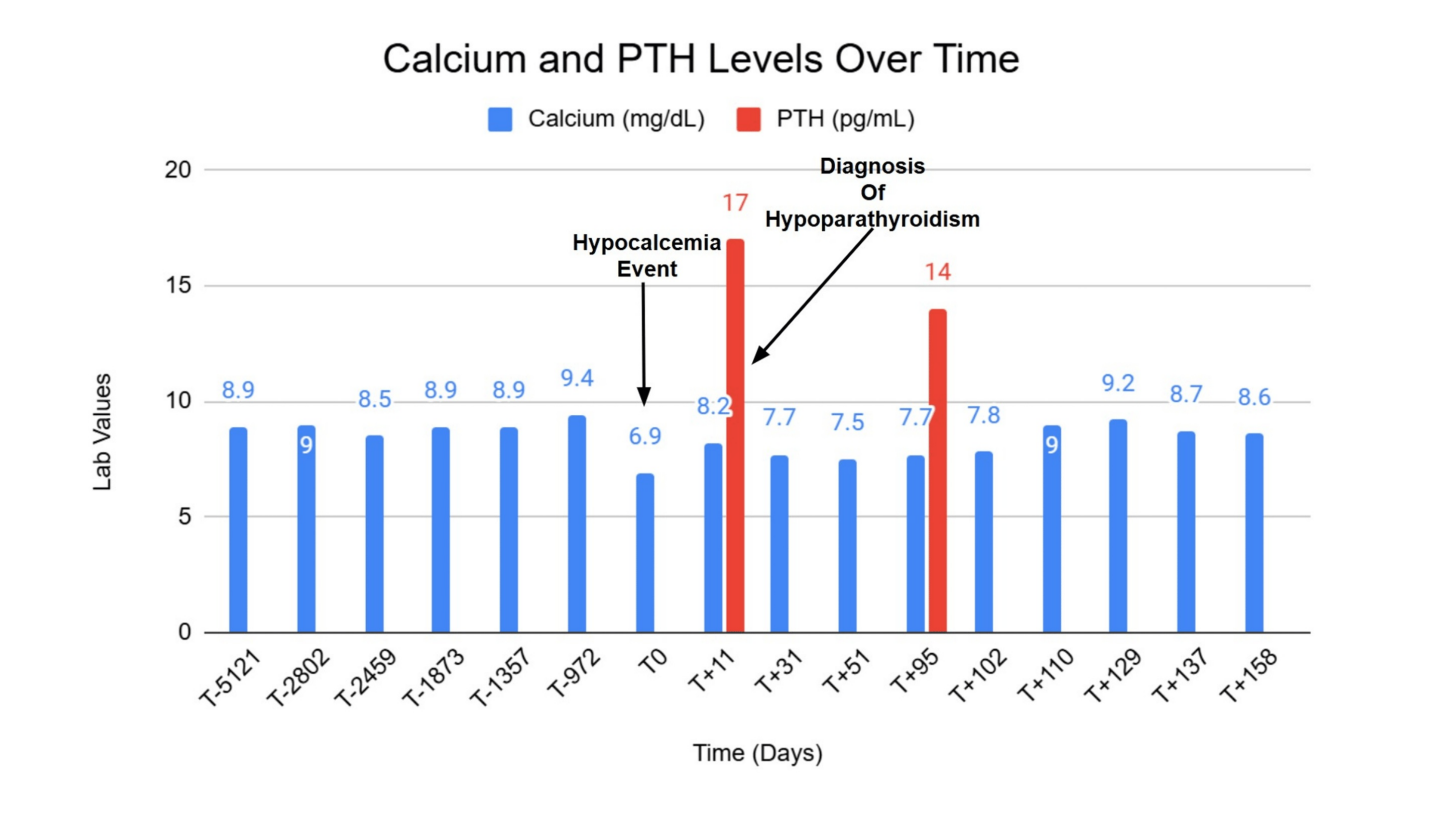
Calcium and PTH levels over time PTH: Parathyroid hormone, T0 denotes date of the hypocalcemic event, T+(n) denotes "n" days after the event, T-(n) denotes "n" days prior to the event

At age 67, repeat DEXA demonstrated a decline in BMD at the hips compared with the prior DEXA, consistent with progression of osteoporosis (Figure 2). Percentage BMD loss was most pronounced at the total hip sites (Figures 2B, 2C), while lumbar spine BMD (Figure 2A) remained relatively stable. Evaluation for secondary causes of osteoporosis at that time again demonstrated normal serum calcium, phosphate, PTH, magnesium, and vitamin D. Based on the DEXA scan results, the treatment plan for the patient was to proceed with denosumab injections. The patient tolerated the injections and subsequently received four doses of denosumab 60 mg subcutaneously every six months without issue until age 69. Throughout this period, the patient’s calcium level remained within the normal range (Figure 3).

Approximately six months after the fourth denosumab dose, routine laboratory testing obtained prior to the fifth denosumab injection revealed hypocalcemia, with a serum calcium level of 6.9 mg/dL (reference range 8.6-10.3 mg/dL). The patient was asymptomatic, denying symptoms of hypocalcemia, including paresthesias, muscle cramps, spasms, seizures, or other cardiac symptoms.

Subsequent blood tests revealed an ionized calcium level of 4.45 mg/dL (4.70-5.90 mg/dL), elevated blood phosphorous of 5.0 mg/dL (2.4-4.6 mg/dL), her albumin-corrected total calcium was 8.2 mg/dL (8.6-10.3 mg/dL), and an inappropriately low PTH level of 17 pg/mL (12-88 pg/mL), along with normal vitamin D 37 ng/mL (30-100 ng/mL) and magnesium 2.1 mg/dL (1.7-2.4 mg/dL) (Table 1). Autoimmune evaluation, including inflammatory markers (ANA, anti-ENA, anti-DNA), was unremarkable, with no clinical features suggestive of autoimmune hypoparathyroidism. Anti-calcium-sensing receptor antibodies were not assessed. Other causes of hypocalcemia were ruled out, including a history of neck surgery, autoimmune disease, magnesium disorders, malabsorptive disorders, genetic disorders, and cardiovascular disorders, before the diagnosis of idiopathic hypoparathyroidism was made. Denosumab was paused, and calcium carbonate 500 mg supplementation (two tabs three times daily) was recommended to the patient.

**Table 1:** Laboratory test results with reference values

Laboratory Test	Value	Reference Range
Ionized calcium	4.45 mg/dL	4.70-5.90 mg/dL
Calcium	8.2 mg/dL	8.6-10.3 mg/dL
Parathyroid hormone	17 pg/mL	12-88 pg/mL
Magnesium	2.1 mg/dL	1.7-2.4 mg/dL
Total vitamin D	37 ng/mL	30-100 ng/mL
Phosphorus	5.0 mg/dL	2.4 -4.6 mg/dL

As calcium levels improved, she was placed back on alendronate 70 mg weekly, despite her previous gastrointestinal issues, to manage her osteoporosis. Pending further workup, the patient was referred to endocrinology. The patient continued calcium carbonate 500 mg twice daily and calcitriol 1.5 mcg daily, resulting in stabilization of serum calcium in the low-normal range over a six-month follow-up period (Figure 3). PTH levels, however, remained suppressed (latest value 14 pg/mL). Renal function remained stable throughout follow-up. Given persistent low PTH levels with low-normal calcium, alternative osteoporosis treatment strategies, including non-antiresorptive options, are currently being evaluated in collaboration with endocrinology.

## Discussion

Idiopathic hypoparathyroidism is a rare diagnosis marked by hypocalcemia, hyperphosphatemia, and reduced 1,25-dihydroxyvitamin D [2,3]. Diagnosis is established by low ionized calcium levels in the presence of undetectable or significantly low PTH levels [2,3]. Idiopathic hypoparathyroidism occurs in a setting of parathyroid gland dysfunction with unclear etiology, often related to an autoimmune process or due to genetic causes [1-3,8]. In idiopathic hypoparathyroidism, PTH levels remain inappropriately low or normal despite hypocalcemia because the parathyroid glands are unable to synthesize or secrete adequate amounts of PTH [1-3,8]. This represents a failure of the normal physiological response, in which hypocalcemia should trigger increased PTH secretion by inactivating the calcium-sensing receptor (CaSR) on parathyroid chief cells [1,8]. In the presence of hypocalcemia, low calcium should stimulate a high PTH; however, PTH is low or on the lower end of normal, which is inappropriate for the degree of hypocalcemia, hence the terminology of "inappropriately low" PTH in idiopathic hypoparathyroidism [1,8].

Given that idiopathic hypoparathyroidism is a rare and uncommon condition, in the setting of denosumab treatment for osteoporosis, it presents unique challenges for osteoporosis management. First, this case highlights the importance of ensuring normal calcium levels before administering denosumab, given the risk of denosumab-induced hypocalcemia. In our patient, hypocalcemia could have worsened significantly with denosumab administration, as denosumab administration is associated with hypocalcemia, particularly in patients with chronic kidney disease [7,8]. The patient’s calcium had previously been normal before each denosumab injection, but hypocalcemia was noted before her most recent denosumab injection. Idiopathic hypoparathyroidism can occur spontaneously and insidiously [2]. In this patient, treatment with denosumab appeared to unmask and worsen hypocalcemia in a patient with pre-existing idiopathic hypoparathyroidism. This occurs because denosumab neutralizes RANKL, preventing osteoclast-mediated bone resorption and removing a compensatory mechanism that maintains serum calcium levels in a system already experiencing calcium dysregulation due to an inability to increase PTH. While denosumab has been associated with hypocalcemia, in general, denosumab administration is expected to increase PTH levels [5]. However, our patient experienced a further decrease in PTH despite having low calcium levels, leading to the uncovering of the patient's diagnosis of idiopathic hypoparathyroidism.

Delayed denosumab injection is associated with a possible increased risk of vertebral compression fractures, as it is associated with increased bone resorption after stopping denosumab [9-12]. The exact etiology is unclear but is believed to occur due to several mechanisms, including an increase in bone turnover markers such as cross-linked C-telopeptide of type I collagen (CTx) coupled with Procollagen Type 1 N-terminal Propeptide (P1NP) above baseline values within 3-6 months after stopping denosumab treatment and an increase in osteoclast precursors that accumulate during denosumab treatment [9-12]. Bisphosphonates have been suggested as a potential option to prevent increased bone resorption [9,10,13]. Bisphosphonates inhibit bone resorption through binding to bone hydroxyapatite and are taken up by osteoclasts, leading to osteoclast apoptosis [9,10,13]. While bisphosphonates can also cause hypocalcemia, the effect is generally less severe and frequent than with denosumab, as they suppress osteoclast activity more gradually and bind to bone, reducing abrupt calcium shifts [7]. Therefore, our patient was taken off denosumab and placed back on bisphosphonate therapy in the interim, while considering other treatment options.

There are no clear guidelines for the treatment of osteoporosis in the setting of idiopathic hypoparathyroidism. Idiopathic hypoparathyroidism is associated with unique bone characteristics, characterized by low bone turnover and a prolonged bone remodeling cycle. However, interestingly, bone mineral density, as measured by DEXA, is above average for age at all sites in hypoparathyroidism [14]. Fracture risk in hypoparathyroidism may not be increased, although this remains unclear due to the rarity of the condition and insufficient experience. Calcium and vitamin D supplementation do not alleviate the skeletal changes noted in hypoparathyroidism. However, the use of PTH (rhPTH(1-84) formulation, approved by the FDA in 2015) has been shown to increase bone mineral density, improve serum calcium levels, and reduce long-term supplemental requirements in patients with idiopathic hypoparathyroidism [15].

For patients with idiopathic hypoparathyroidism and hypocalcemia, calcium and 1,25-dihydroxyvitamin D supplementation is indicated to maintain calcium levels in the low-normal reference range of 8-8.5 mg/dL while minimizing hypercalciuria and ectopic calcification. Thiazide diuretics can be added to increase renal calcium reabsorption and control excess urinary calcium excretion in patients with hypercalciuria when necessary. Teriparatide and rhPTH(1-84) have been studied as adjunctive or replacement options in the treatment of hypoparathyroidism. Specifically, rhPTH (1-84) is now FDA-approved for patients who cannot be well-controlled on conventional therapy alone and can reduce the need for supplemental calcium and active vitamin D [16]. However, the use of this treatment and its efficacy in the management of idiopathic hypoparathyroidism specifically are unclear due to a lack of evidence and studies to document this. In this patient’s case, calcium and vitamin D supplementation restored their calcium levels to the low-normal range.

This study had several limitations, including limited follow-up time due to the case being recent, a lack of bone biopsy, and a lack of extensive genetic workup, including gene sequencing. Future studies are needed to investigate the safety of denosumab in hypoparathyroidism and to define optimal osteoporosis management strategies in this setting. Additionally, future studies could investigate the role of recombinant PTH therapy for the management of both hypoparathyroidism and osteoporosis.

## Conclusions

In summary, we present a rare case of idiopathic hypoparathyroidism during the treatment of osteoporosis. This case underscores the importance of routinely checking and correcting serum calcium levels prior to denosumab administration, particularly in elderly patients and those at risk for disorders of calcium homeostasis. It also raises the question of whether targeted screening for hypoparathyroidism should be considered before initiating potent antiresorptive therapy in patients with unexplained hypocalcemia or low-normal calcium levels. Osteoporosis management in idiopathic hypoparathyroidism requires careful attention to baseline calcium, vitamin D status, and close biochemical monitoring predating and following treatment. This case highlights the need for future studies to inform evidence-based osteoporosis treatment algorithms and guideline development for patients with idiopathic hypoparathyroidism.
